# Secondary Metabolites from the Roots of *Beilschmiedia tsangii* and Their Anti-Inflammatory Activities

**DOI:** 10.3390/ijms131216430

**Published:** 2012-12-03

**Authors:** Yun-Ting Huang, Hsun-Shuo Chang, Guei-Jane Wang, Chu-Hung Lin, Ih-Sheng Chen

**Affiliations:** 1 Graduate Institute of Natural Products, College of Pharmacy, Kaohsiung Medical University, Kaohsiung 80708, Taiwan; E-Mails: leave.alone@hotmail.com (Y.-T.H.); hschang@kmu.edu.tw (H.-S.C.); 2 L5 Research Center, China Medical University Hospital, Taichung 40447, Taiwan; E-Mail: jennyw355@gmail.com; 3 Graduate Institute of Clinical Medical Science, China Medical University, Taichung 40402, Taiwan; 4 School of Pharmacy, College of Pharmacy, Kaohsiung Medical University, Kaohsiung 80708, Taiwan; E-Mail: u96830006@kmu.edu.tw

**Keywords:** *Beilschmiedia tsangii*, Lauraceae, root, endiandric acid, anti-inflammatory activity

## Abstract

Four new endiandric acid analogues, tsangibeilin C (**1**), tsangibeilin D (**2**), tricyclotsangibeilin (**3**) and endiandric acid M (**4**), one new lignan, beilschminol B (**5**) and two new sesquiterpenes, (+)-5-hydroxybarbatenal (**6**) and (4*R*,5*R*)-4,5-dihydroxycaryophyll-8(13)-ene (**7**), together with four known compounds (**8**–**11**), were isolated from the roots of *Beilschmiedia tsangii* (Lauraceae). The structures of **1**–**7** were determined by spectroscopic techniques. Among the isolates, endiandric acid M (**4**) exhibited moderate iNOS inhibitory activity, with an IC_50_ value of 31.70 μM.

## 1. Introduction

**Figure 1:**
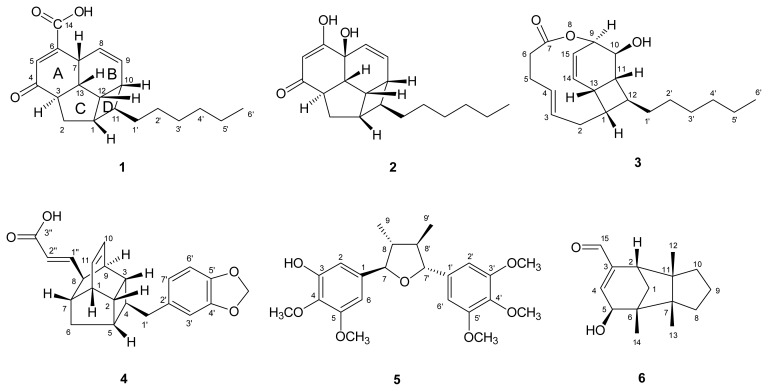

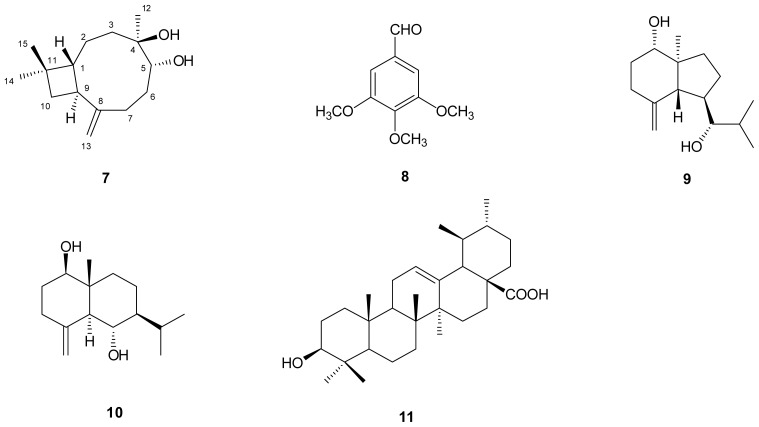
Structures of compounds **1**–**11**.

Over 40 species of Formosan lauraceous plants have been screened for anti-inflammatory activity using an inducible nitric oxide synthase (iNOS) assay. A methanolic extract of the roots of *B. tsangii* Merr. (Lauraceae) has shown potent inhibition of NO production, with no cytotoxicity against RAW 264.7 cells. *B. tsangii* is a medium-sized evergreen tree, distributed throughout Tonkin, Vietnam, southern and western China and southern Taiwan. About 200 Beilschmiedia species are found in these tropical regions, including two species in Taiwan [1]. Our previous study reported two new tetrahydrofuran-type lignans, beilschmin A and beilschmin B, two new 1-phenylbutyl benzoates, tsangin A and tsangin B, together with thirteen known compounds isolated from the stem of this plant [2]. One year later, three new epoxyfuranoid lignans, 4α,5α-epoxybeilschmin A, 4α,5α-epoxybeilschmin B and beilschmin D, together with nine known compounds, were obtained from the leaves [3]. More recently, six new endiandric acid analogues, tsangibeilin A, tsangibeilin B, endiandramide A, endiandric acid K, endiandric acid L and endiandramide B, two new lignans, beilschminol A and tsangin C, and six known compounds have been obtained from the roots of this species [4]. In this continuation of our research, four new endiandric acid analogues, tsangibeilin C (**1**), tsangibeilin D (**2**), tricyclotsangibeilin (**3**) and endiandric acid M (**4**), one new lignan, beilschminol B (**5**) and two new sesquiterpenes: (+)-5-hydroxybarbatenal (**6**) and (4*R*,5*R*)-4,5-dihydroxycaryophyll-8(13)-ene (**7**), together with four known compounds (**8**–**11**), have been isolated from the roots of this plant. The structures of **1**–**7** were determined by spectroscopic techniques. This paper elucidates the structures of **1**–**7** (Figure 1). Endiandric acid M (**4**) exhibited moderate iNOS inhibitory activity, with an IC_50_ value of 31.70 μM.

## 2. Results and Discussion

### 2.1. Structure Elucidation

**Figure 2:**
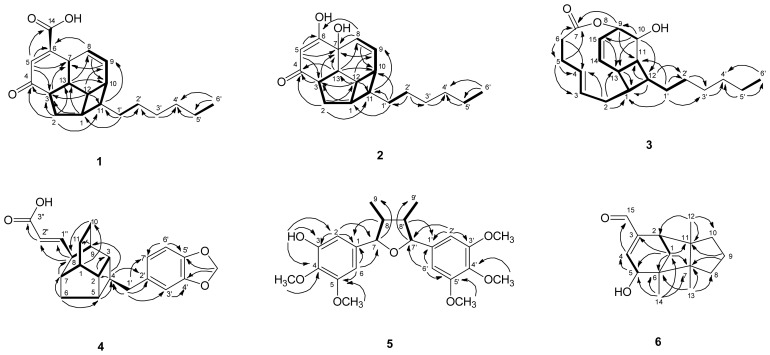

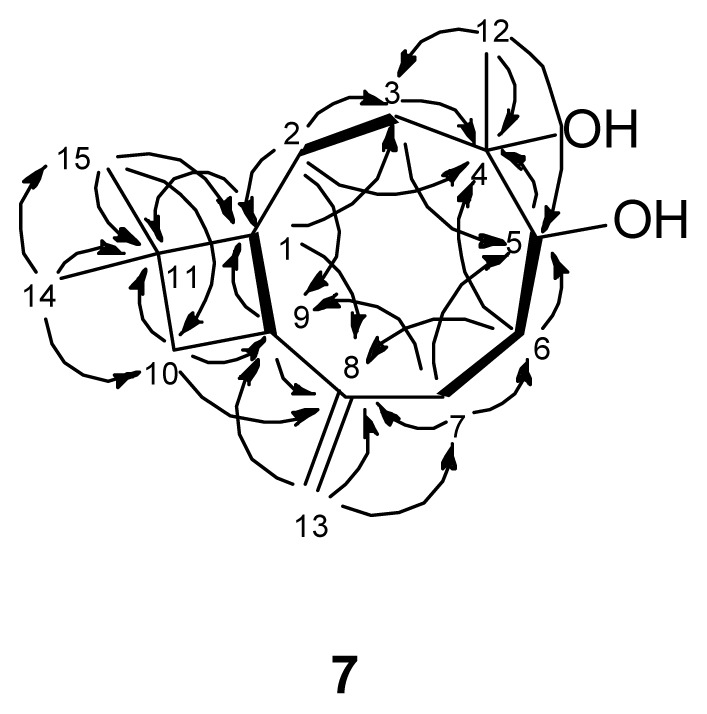
COSY (**——**) and HMBC ( 
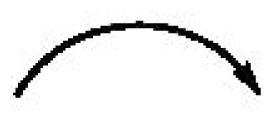
) connectivities for compounds **1**–**7**.

**Figure 3:**
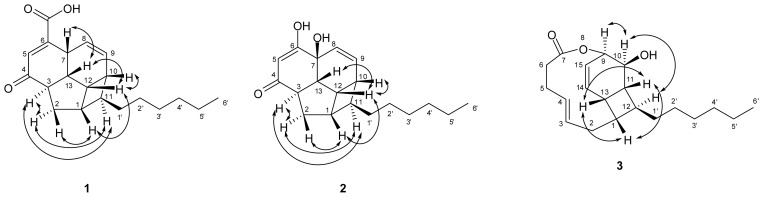

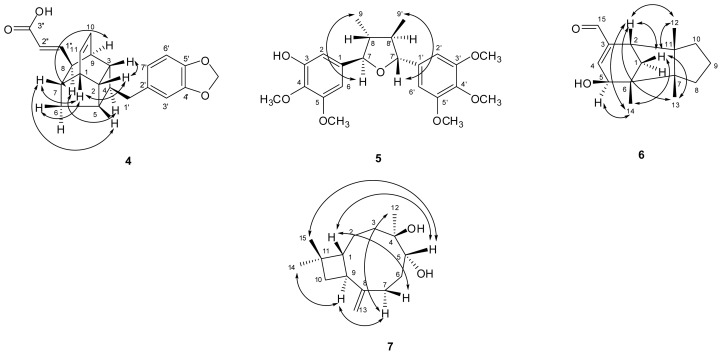
NOESY ( 
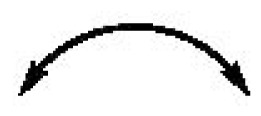
) connectivities for compounds **1**–**7**.

Compound **1** was isolated as a light yellowish oil. Its molecular formula was established as C_20_H_26_O_3_ by ESIMS and HRESIMS, with eight unsaturated degrees. The IR spectrum showed absorptions at 1696 cm^−1^ for carbonyl groups (*δ* 201.6, C-4 and *δ* 168.3, C-14) and at 3432 cm^−1^ for a hydroxy group of carboxylic acid. These findings were supported by ^13^C NMR spectrum. The ^1^H, ^13^C NMR (Table 1), COSY (Figure 2), HSQC and HMBC (Figure 2) spectra of **1** were similar to those of beilschmiedic acid D [5] and also contained 13 skeletal signals of an endiandric acid moiety. The characteristic two *cis* olefinic protons at δ 5.56 (ddd, *J =* 10.2, 3.0, 1.8 Hz, H-8) and 5.85 (ddd, *J =* 10.2, 4.2, 3.0 Hz, H-9) in **1** were similar to those of beilschmiedic acid D, but the signal for another olefinic proton in **1** was shifted upfield to δ 6.70 (d, *J =* 1.2 Hz, H-5), because a carbonyl group (δ_C_ 201.6, C-4) in **1** replaced a methylene group [δ 2.06 (m, H_a_-4) and 2.54 (dt, *J =* 8.8, 3.1 Hz, H_b_-4)] in beilschmiedic acid D. The length of the alkyl side chain at C-11 of **1** was two methylenes less than beilschmiedic acid D, as supported by the molecular formula of **1** (C_20_H_26_O_3_). The rigid tetracyclic skeleton was indicated by HMBC correlations, including: H-5 to C-3, C-6, C-7 and C-14, H-3 to C-4, and C-7, H-13 to C-8 and C-10, H-8 to C-6 and C-10, H-9 to C-7, H-2 to C-3, C-4, C-11 and C-13, H-1 to C-3 and C-13, H-12 to C-3, C-9 and C-11 and H-11 to C-9. The relative configuration of **1**, *rel*-(1*S*,3*R*,7*R*,10*R*,11*S*,12*S*,13*S*), was supported on the basis of NOESY (Figure 3) and structural similarity with beilschmiedic acid D [5]. There are seven chiral centers in **1**. However, in view of the observed optical rotation ([α]_23_^D^ ± 0), **1** should be racemic, as are many endiandric acid analogues [6–9]. According to the above data, the structure of **1** was elucidated and named tsangibeilin C.

Analysis of the ESIMS and HREIMS of **2** revealed a molecular formula of C_19_H_26_O_3_, representing seven degrees of unsaturation, one carbon and one degree of unsaturation less than compound **1** (C_20_H_26_O_3_). The IR spectrum indicated the presence of a hydroxy group (3397 cm^−1^) and a carbonyl group (1692 cm^−1^). The rigid tetracyclic skeleton of **2** was the same as that of **1**, according to its ^1^H and ^13^C NMR spectra (Table 1), inclusive of COSY (Figure 2), NOESY (Figure 3), HSQC and HMBC (Figure 2) experiments. The major differences between **1** and **2** were two hydroxy groups at C-6 and C-7 in **2** were substituted for the carboxyl group (*δ* 168.3, C-14) and H-7 [δ_H_ 3.51 (1H, br s)] at C-6 and C-7 in **1**, as supported by HRESIMS, IR and DEPT spectra. The NOESY spectrum (Figure 3) showed correlations between H_a_-2, H-3 and H-11, but these three protons showed no correlations with H-1, H_b_-2, H-10, H-12 and H-13. This suggested that H_a_-2, H-3 and H-11 are on the same side of the molecule, and that H-1, H_b_-2, H-10, H-12 and H-13 are on the opposite side of the molecule. The β-orientation of the hydroxy group at C-7 was attributed according to the structural similarity with endiandric acid analogues and biogenetic consideration, where the rings A/B, B/C, C/D and B/D were *cis*-fusion and ring A/C was *trans*-fusion [4,8,9]. The relative configuration of **2**, *rel*-(1*S*,3*R*,7*R*,10*R*,11*S*,12*S*,13*S*) was supported on the basis of NOESY and structural similarity with beilschmiedic acid D [5]. On the basis of the observations above, compound **2** was elucidated and named tsangibeilin D.

Compound **3** was obtained as an optically active light yellowish oil with [α]_24_^D^ + 36.3 (*c* 0.024, CHCl_3_). IR absorption bands at 3422 cm^−1^ (OH) and 1729 cm^−1^ (ester carbonyl) were observed. The ESIMS analysis of **3** showed the [M+Na]^+^ ion at *m/z* 341, in agreement with the molecular formula of C_20_H_30_O_3_, with six degrees of unsaturation as confirmed by HRESIMS. The ^13^C NMR (Table 2) and DEPT spectra indicated that **3** contains one methyl, eight methylenes, ten methines and one quaternary carbon. The HSQC and COSY (Figure 2) spectra revealed three fragments, C1-C2-C3-C4-C5-C6, C1-C13-C11-C12-C1 and C-13-C14-C15-C9-C10, and the HMBC (Figure 2) correlations, H-10 to C-9, C-12, C-13 and C-15 and H-9 to C-11, connected the fragments C1-C13-C11-C12-C1 and C-13-C14-C15-C9-C10 to form a cyclohexane ring fused with a cyclobutane ring. The carboxyl group (*δ* 173.5, C-7) connected the fragment C1-C2-C3-C4-C5-C6 with the cyclohexane ring, as supported by HMBC correlations, H-6 to C-4, C-5 and C-7, H-5 to C-3 and C-7 and H-9 to C-7. According to the above evidence, the carboxyl group, the fragment C1-C2-C3-C4-C5-C6, the cyclohexane ring and the cyclobutane ring become a cyclododecane ring, which is also supported by six degrees of unsaturation. The HMBC spectrum showed correlations between H-1′ and C-1, C-11, C-2′ and C-3′, thus establishing the presence of an alkyl chain (C-1′~C-6′) to be connected at C-12. Finally, the HRESIMS, IR, and DEPT spectra indicated the presence of a hydroxy group located at C-10. The NOESY spectrum (Figure 3) showed correlations between H-9, H-10 and H-12, suggesting these three protons were on the same side of the molecule, but H-1, H-11 and H-13 showed no correlations with H-9, H-10 and H-12, which should therefore be on the opposite side of the molecule. The relative configuration of **3**, *rel*-(1*S*,9*R*,10*S*,11*R*,12*S*,13*S*), was supported on the basis of NOESY and its structural similarity with know endiandric acid analogues. Based on further spectrographic evidence, the structure of **3** was elucidated and designated as tricyclotsangibeilin.

ESIMS and HRESIMS data of compound **4** indicated the molecular formula C_22_H_22_O_4_, four methylenes less than endiandric acid L [4] with 12 degrees of unsaturation, which was the same as endiandric acid L. The UV, IR, ^1^H NMR (Table 2) and ^13^C NMR (Table 2) spectra were similar to those of endiandric acid L, except that a methylene group [δ 2.78 (1H, dd, *J* = 15.6, 7.8 Hz, H_a_-1′), 2.81 (1H, br d, *J* = 7.8 Hz, H_b_-1′)] in **4** replaced the pentamethylene group in endiandric acid L. The relative configuration of **4** was supported by NOESY spectrum and its structural similarity with endiandric acid L [4]. Thus, compound **4** was named endiandric acid M. Compounds **1**, **2** and **4** were racemic, and all structures were determined by ^13^C NMR, NOESY, COSY, HSQC and HMBC experiments (see Figures 2 and 3).

Compound **5** was obtained as an optically active light yellowish oil, [α]_25_^D^ –18.6 (*c* 0.18, MeOH), and its molecular formula was established as C_23_H_30_O_7_ by ESIMS and HRESIMS. UV absorptions at 209, 240 sh, and 270 nm, in conjunction with a bathochromic shift after the addition of alkali, indicated a phenolic benzenoid moiety. An IR absorption band at 3415 cm^−1^ (OH) was also observed. The ^1^H NMR signals for a 7,7′-epoxylignan moiety [δ 1.07 (3H, d, *J* = 6.0 Hz, H-9′), 1.08 (3H, d, *J =* 6.0 Hz, H-9), 1.78 (1H, m, H-8), 1.78 (1H, m, H-8′), 4.61 (1H, d, *J* = 8.8 Hz, H-7) and 4.63 (1H, d, *J* = 9.2 Hz, H-7′)] were similar to those of beilschminol A [4], except that OCH_3_-3′ [δ 3.88 (3H, s)] and OCH_3_-4′ [δ 3.83 (3H, s)] in **5** replaced a methylenedioxy group [δ 5.96 (2H, each d, *J =* 1.4 Hz)] in beilschminol A. Thus, the planar structure of **5** was proposed to be 3-hydroxy-4,5,3′,4′,5′-pentamethoxy-7,7′-epoxylignan. The relative configuration of **5** was based on the NOESY spectrum and its structural similarity with (7*R*,8*R*,7′*R*,8′*R*)-3-hydroxy-3′,4′-methylenedioxy-4,5,5′-trimethoxy-7,7′-epoxylign [4]. Thus, compound **5** was elucidated as *rel*-(7*R*,8*R*,7′*R*,8′*R*)-3-hydroxy-4,5,3′,4′,5′-pentamethoxy-7,7′-epoxylignan and named beilschminol B.

Compound **6** was isolated as an optically active light yellowish oil, [α]_25_^D^ + 123.8 (*c* 0.06, CHCl_3_). The HRESIMS analysis of **6** gave a quasi-molecular ion peak at *m/z* 257.1516 [M+Na]^+^ (calcd. for C_15_H_22_O_2_Na, 257.1517), consistent with a molecular formula of C_15_H_22_O_2_ with five degrees of unsaturation. The IR, ^1^H, and ^13^C NMR (Table 3) spectra resembled those of (+)-barbatenal [10], except that a hydroxy group and an oxymethine proton [*δ* 4.09 (1H, d, *J* = 3.6, H-5)] in C-5 (*δ*_c_ 71.2) of **6** replaced a methylene [*δ* 1.65 (1H, dd, *J =* 21.1, 3.3 Hz, H_a_-5), 2.04 (1H, dd, *J =* 20.1, 2.8 Hz, H_b_-5)] in C-5 (*δ*_c_ 41.9) of (+)-barbatenal, resulting in the downshifting of the olefinic proton (*δ*_H_ 6.60, H-4) in **6** from *δ*_H_ 5.87 (H-4) in (+)-barbatenal. The relative configuration of **6** was determined by NOESY spectrum, where H_a_-1 showed correlations with H-2, H-12, H-13 and H-14, but no correlation with H-5. It has been suggested that H_a_-1, H-2, H-12, H-13 and H-14 are on the same side of the molecule and that H_b_-1 and H-5 are on the opposite side of the molecule. Furthermore, H-5 showed a correlation with H-14 due to a spatial distance between these two protons of <4 Å, as supported by simulating the ChemDraw 3D Ultra (version 10.0). Based on the NOESY spectrum, the optical rotation and comparison to values in the literature, the relative configuration of **6** was determined to be *rel*-(2β, 5α, 12β, 13β, 14β). Hence, the structure of **6** was elucidated and designated as (+)-5-hydroxybarbatenal, which was further confirmed by DEPT, HSQC, NOESY (Figure 3) and HMBC (Figure 2) techniques.

Compound **7** was isolated as an optically active light yellowish oil with [α]_25_^D^ –23.9 (*c* 0.032, CHCl_3_). IR absorption bands at 3396 cm^−1^ (OH) and 1637 cm^−1^ (C=C) were observed. From the HRESIMS data, the molecular formula was determined to be C_15_H_26_O_2_ (*m/z* 261.1828 [M+Na]^+^; calcd. 261.1830). The ^13^C NMR (Table 3) and DEPT spectra indicated that **7** contains three methyls, six methylenes, three methines and three quaternary carbons, suggesting a sesquiterpene skeleton. The HSQC and COSY (Figure 2) spectra revealed the correlations from C1-C9, C2-C3 and C-5-C6-C7 and the HMBC (Figure 2) correlations, H-1 to C-3, C-8 and C-11, H-10 to C-8, C-9 and C-11, H-9 to C-1 and C-8, H-7 to C-5, C-6, C-8 and C-9, H-6 to C-4, C-5 and C-8, H-3 to C-4 and C-5 and H-2 to C-1, C-3, C-4 and C-9, connected the fragments C1-C9, C2-C3 and C-5-C6-C7 to form a cyclononane ring and a cyclobutane ring. HMBC correlations, H-15 to C-1, C-10 and C-11, H-14 to C-10, C-11 and C-15, H-12 to C-3, C-4 and C-5, H-13 to C-7, C-8 and C-9, confirmed that three methyl groups were connected at C-4 and C-11 and that a terminal double bond was present at C-8. Finally, the HRESIMS, IR, DEPT spectra indicated two hydroxy groups located at C-4 and C-5, respectively. The NOESY spectrum (Figure 3) showed correlations between H_b_-7, H-9, H-12 and H-14, suggesting these three protons were on the same side of the molecule, but H-1, H-5, H_a_-7 and H-15 showed no correlations with H_b_-7, H-9, H-12 and H-14 and, therefore, should be on the opposite side of the molecule. The structure of **7** was elucidated as (4*R*, 5*R*)-4,5-dihydroxycaryophyll-8(13)-ene and identified with its synthetic compound [11]. Though compound **7** has previously been reported as a reduction intermediate [11], our study is the first time to have isolated this compound from a natural source.

The known compounds, 3,4,5-trimethoxybenzaldehyde (**8**) [12], octahydro-4-hydroxy-3α-methyl-7-methylene-α-(1-methylethyl)-1*H*-indene-1-methanol (**9**) [13,14], eudesm-4(15)-ene-1β,6α-diol (**10**) [15,16] and ursolic acid (**11**) [17] were identified by comparison of their physical and spectroscopic data with values reported in the literature.

### 2.2. Anti-inflammatory Activities

E_max_ (%) and IC_50_ (μM) values of iNOS inhibitory activity of five compounds, including compounds **4**, **5** and **11**, and the two compounds, 6β-hydroxystigmast-4-en-3-one and *rel*-(7*S*,8*S*,7′*R*,8′*R*)-3,3′,4,4′,5,5′-hexmethoxylignan, reported by Huang *et al.*, 2011 [4], were obtained at the concentration range of 0.1 to 100 μM. Results are shown in Table 4. The technique for the anti-iNOS activity assay was the same as in our previous study [4]. Among the isolates, endiandric acid M (**4**) exhibited moderate iNOS inhibitory activity, with IC_50_ value of 31.70 μM. Endiandramide B [4], endiandric acids K [4], L [4] and M, with the same skeleton but with different substituents at C-8, exhibited ascending degrees of iNOS inhibitory activity in this order: endiandramide B (16.40 μM) > endiandric acid M (**4**) (31.70 μM) > endiandric acid L (39.56 μM) > endiandric acid K (58.21 μM). This suggests that the potency of the substituent at C-8 was an *N*-isobutylamido group > an α,β-unsaturated carboxylic acid group > a carboxylic acid group. Endiandric acid M (**4**), with four fewer methylenes than endiandric acid L, showed stronger potency than endiandric acid L. This suggests that fewer methylenes in the alkyl side of endiandric acid analogues result in greater potency of iNOS inhibitory activity.

## 3. Experimental Section

### 3.1. General Experimental Procedures

All melting points were measured on a Yanaco micro-melting apparatus and were uncorrected. Optical rotations were measured on a Jasco P-1020 digital polarimeter. The UV spectra were obtained with a Jasco V-530 UV/VIS spectrophotometer, and IR spectra (KBr or neat) were taken on a Perkin-Elmer System 2000 FT-IR spectrometer. 1D (^1^H, ^13^C, DEPT) and 2D (COSY, NOESY, HSQC, HMBC) NMR spectra using CDCl_3_ or acetone-*d_6_* or CD_3_OD as solvent were recorded on Varian Germini 2000-200 (200 MHz for ^1^H NMR, 50 MHz for ^13^C NMR), Varian Unity Plus 400 (400 MHz for ^1^H NMR, 100 MHz for ^13^C NMR) and Varian VNMRS-600 (600 MHz for ^1^H NMR, 150 MHz for ^13^C NMR) spectrometers. Chemical shifts were internally referenced to the solvent signals in CDCl_3_ (^1^H, *δ* 7.26; ^13^C, *δ* 77.0) or acetone-*d_6_* (^1^H, *δ* 2.05; ^13^C, *δ* 205.1) or CD_3_OD (^1^H, *δ* 3.31; ^13^C, *δ* 49.0), with TMS as the internal standard. ESIMS were obtained on an API 3000 mass spectrometer (Applied Biosystems) and HRESIMS on a Bruker Daltonics APEX II 30e mass spectrometer. Silica gels (70–230, 230–400 mesh) (Merck) were used for column chromatography (CC), and silica gel 60 F-254 (Merck) was used for analytical and preparative TLC. A spherical C18 100A column (20–40 μM) (Silicycle) was used for medium-pressure liquid chromatography.

### 3.2. Plant Material

Roots of *B. tsangii* were collected at Mudan, Pingtung County, Taiwan, in April 2009 and identified by one of the authors (I.-S.C.). A voucher specimen (Chen 6120) was deposited in the Herbarium of the School of Pharmacy, College of Pharmacy, Kaohsiung Medical University, Kaohsiung, Taiwan, Republic of China.

### 3.3. Extraction and Isolation

The dried roots (7.9 kg) of *B. tsangii* were sliced and extracted three times with cold MeOH (10 L) at room temperature. The concentrated MeOH extract (280 g) inhibited nitrite production below 25%, with no observed cytotoxicity at 100 μg/mL. The MeOH extract was partitioned using EtOAc-H_2_O (1:1) to obtain EtOAc-soluble (120 g) and H_2_O-soluble (80 g) fractions. The EtOAc solubles (100 g) were applied to a silica gel CC and eluted with *n*-hexane-EtOAc gradient solvent system (15:1 to 100% EtOAc) to obtain 13 fractions (A-1–A-13). Fractions A-4–A-13 showed inhibition of nitrite production using the anti-iNOS assay, and the H_2_O-soluble fraction showed no inhibitory activity. CC of fraction A-8 (9.6 g) on silica gel, eluting with a gradient of CH_2_Cl_2_–EtOAc, yielded fractions A-8-1 to A-8-10. Fraction A-8-5 (416 mg) was subjected to MPLC (RP-18), eluting with acetone:H_2_O (3:2), to obtain fractions A-8-5-1 to A-8-5-14. Fraction 8-5-6 (11.7 mg) was applied to preparative TLC (*n*-hexane:acetone, 3:1) to obtain **6** (2.8 mg). Fraction A-8-5-14 (210 mg) was applied to MPLC (RP-18) eluting with acetone:H_2_O (2:1) to give 20 fractions (A-8-5-14-1–A-8-5-14-20). Fraction A-8-5-14-10 (20.8 mg) was separated over MPLC eluting with *n*-hexane:acetone (8:1) to afford **3** (1.2 mg). Fraction A-8-7 (1.97 g) was submitted to MPLC (RP-18), eluting with MeOH:H_2_O (10:1), to obtain fractions A-8-7-1 to A-8-7-14. Fraction A-8-7-6 (223 mg) was applied to MPLC and eluted with *n*-hexane: acetone (4:1) to obtain nine fractions (A-8-7-6-1–A-8-7-6-9). Fraction A-8-7-6-7 (5.5 mg) was purified by preparative TLC (RP-18) developed with MeOH:H_2_O (15:1) to give **4** (4.4 mg). Fraction A-8-7-11 was subjected to MPLC and eluted with *n*-hexane: acetone (5:1) to yield **11** (5.7 mg). Fraction A-9 (17.8 g), on silica gel CC, eluting with a gradient of *n*-hexane–acetone, provided fractions A-9-1 to A-9-16. Fraction A-9-4 (104 mg) was applied to MPLC (RP-18) and eluted with acetone:H_2_O (1:1) to provide **7** (1.6 mg) and 15 fractions (A-9-4-1–A-9-4-15). Fraction A-9-4-12 (3.6 mg) was purified by preparative TLC (RP-18) and developed with *n*-hexane:EtOAc (2:1) to give **10** (3.2 mg). Fraction A-9-4-4 was treated with the same step as fraction A-9-4-12 to yield **9** (1.1 mg). Fraction A-9-9 (587 mg) was separated over MPLC (RP-18), eluting with a system of acetone:H_2_O (1:1), to afford fractions A-9-9-1 to A-9-9-21. Fraction A-9-9-2 (5 mg) and fraction A-9-9-10 (13.3 mg) were applied by preparative TLC developed with CH_2_Cl_2_:MeOH (70:1) and CH_2_Cl_2_:EtOAc (8:1) to give **8** (2.5 mg) and **5** (8.9 mg), respectively. Fraction A-9-9-20 (462 mg) was submitted to MPLC (RP-18) and eluted with acetone:H_2_O (3:1) to afford fractions A-9-9-20-1 to A-9-9-20-15. Fraction A-9-9-20-6 (63.8 mg) was subjected to MPLC (RP-18) and eluted with acetone:H_2_O (2:1) to afford fractions A-9-9-20-6-1 to A-9-9-20-6-12. Fraction A-9-9-20-6-3 (3.8 mg) and fraction A-9-9-20-6-7 (13.3 mg) were purified by preparative TLC (RP-18), developed with MeOH:H_2_O (5:1) and acetone: H_2_O (2:1), to give **1** (3.7 mg) and **2** (3.5 mg), respectively.

Tsangibeilin C (**1**): Light yellowish oil; [α]_23_^D^ ±0 (*c* 0.07, CHCl_3_); IR (neat) ν_max_ cm^−1^: 3432 (hydroxy group of carboxylic acid), 1696 (C=O); for ^1^H and ^13^C NMR spectroscopic data, see Table 1; ESIMS: *m*/*z* 337 [M + Na]^+^; HRESIMS: *m*/*z* 337.1782 [M + Na]^+^ (calcd. for C_20_H_26_O_3_Na, 337.1780).

Tsangibeilin D (**2**): Light yellowish oil; [α]_24_^D^ ±0 (*c* 0.07, CHCl_3_); IR (neat) ν_max_ cm^−1^: 3397 (OH), 1692 (C=O); for ^1^H and ^13^C NMR spectroscopic data, see Table 1; ESIMS: *m*/*z* 325 [M + Na]^+^; HREIMS: *m*/*z* 302.1879 [M]^+^ (calcd. for C_19_H_26_O_3_, 302.1882).

Tricyclotsangibeilin (**3**): Light yellowish oil; [α]_24_^D^ + 36.3 (*c* 0.024, CHCl_3_); IR (neat) ν_max_ cm^−1^: 3422 (OH), 1729 (C=O); for ^1^H and ^13^C NMR spectroscopic data, see Table 2; ESIMS: *m*/*z* 341 [M + Na]^+^; HRESIMS: *m/z* 341.2096 [M + Na]^+^ (calcd. for C_20_H_30_O_3_Na, 341.2093).

Endiandric acid M (**4**): Light yellowish oil; [α]_25_^D^ ±0 (*c* 0.09, CHCl_3_); IR (neat) ν_max_ cm^−1^: 3461 (hydroxy group of carboxylic acid), 1694 (C=O), 1040, 937 (OCH_2_O); for ^1^H and ^13^C NMR spectroscopic data, see Table 2; ESIMS: *m*/*z* 373 [M + Na]^+^; HRESIMS: *m*/*z* 373.1418 [M + Na]^+^ (calcd. for C_22_H_22_O_4_Na, 373.1416).

Beilschminol B (**5**): Light yellowish oil; [α]_25_^D^ –18.6 (*c* 0.18, MeOH); UV (MeOH) λmax (log ɛ): 209 (4.63), 240 sh (3.92), 270 (3.12) nm; UV (MeOH+KOH) λmax (log ɛ): 210 (4.71), 242 sh (3.97), 280 (3.25) nm; IR (neat) ν_max_ cm^−1^: 3415 (OH), 1593, 1508, 1461 (benzene ring); ^1^H NMR (400 MHz, CDCl_3_) *δ* 1.07 (3H, d, *J =* 6.0 Hz, H-9′), 1.08 (3H, d, *J =* 6.0 Hz, H-9), 1.78 (2H, m, H-8, H-8′), 3.83 (3H, s, OCH_3_-4′), 3.88 (12H, s, OCH_3_-3′, OCH_3_-4, OCH_3_-5, OCH_3_-5′), 4.61 (1H, d, *J =* 8.8 Hz, H-7), 4.63 (1H, d, *J =* 9.2 Hz, H-7′), 5.78 (1H, br s, D_2_O exchangeable, OH-3), 6.55 (1H, d, *J =* 1.8 Hz, H-6), 6.61 (2H, s, H-2′, H-6′), 6.64 (1H, d, *J =* 1.8 Hz, H-2); ^13^C NMR (100 MHz, CDCl_3_) *δ* 13.9 (C-9′), 14.0 (C-9), 50.9 (C-8′), 51.0 (C-8), 55.9 (OCH_3_-5), 56.1 (OCH_3_-3′, OCH_3_-5′), 60.8 (OCH_3_-4′), 60.9 (OCH_3_-4), 88.2 (C-7), 88.5 (C-7′), 101.6 (C-6), 102.9 (C-2′, C-6′), 105.8 (C-2), 134.7 (C-4), 137.2 (C-4′), 138.0 (C-1′), 138.7 (C-1), 149.1 (C-3), 152.4 (C-5), 153.2 (C-3′, C-5′); ESIMS: *m*/*z* 441 [M + Na]^+^; HRESIMS: *m*/*z* 441.1887 [M + Na]^+^ (calcd. for C_23_H_30_O_7_Na, 441.1889).

(+)-5-Hydroxybarbatenal (**6**): Light yellowish oil; [α]_25_^D^ +123.8 (c 0.06, CHCl_3_); IR (neat) ν_max_ cm^−1^: 3408 (OH), 1681 (C=O); for ^1^H and ^13^C NMR spectroscopic data, see Table 3; ESIMS: *m*/*z* 257 [M + Na]^+^; HRESIMS: *m*/*z* 257.1516 [M + Na]^+^ (calcd. for C_15_H_22_O_2_Na, 257.1517).

(4*R*,5*R*)-4,5-Dihydroxycaryophyll-8(13)-ene (**7**): Light yellowish oil; [α]_25_^D^ –23.9 (c 0.032, CHCl_3_); IR (neat) ν_max_ cm^−1^: 3396 (OH), 1637 (C=C); for ^1^H and ^13^C NMR spectroscopic data, see Table 3; ESIMS: *m*/*z* 261 [M + Na]^+^; HRESIMS: *m*/*z* 261.1828 [M + Na]^+^ (calcd. for C_15_H_26_O_2_Na, 261.1830).

## 4. Conclusions

There are about 200 Beilschmiedia species in the tropical regions mentioned earlier, including two species in Taiwan [1]. Except for the two species in Taiwan, previous studies of nine *Beilschmiedia* species in the world have revealed endiandric acids [5,18,19], flavonoids [20,21], alkaloids [22–24], benzopyrans [18], arylpropanoids [25] and sesquiterpenoids [18]. Several isolates have shown antibacterial [19,21] and antimalarial [22] activities. Phytochemical studies of the two Formosan Beilschmiedia species have been carried out by our group, analyzing the stems [2], the leaves [3] and the roots [4] of *B. tsangii,* together with the roots [8,9] of *B. erythrophloia*. Both species possess endiandric acids, 7,7′-epoxylignans, 7′,8′-seco-7,7′-epoxylignans, benzopyrans, arylpropanoids, amides, sesquiterpenoids and steroids. In this study, endiandric acids, 7,7′-epoxylignans, benzenoid, sesquiterpenoids and triterpenoid were also reported. The constituents of the roots of the two Formosan Beilschmiedia species showed 19 new endiandric acid analogues as key secondary metabolites. The roots of other Beilschmiedia species found in other regions have not yet been studied. However, several isolates from two Formosan Beilschmiedia species have shown cytotoxic [2], anti-tubercular [3,9] or anti-inflammatory activities [4] in previous studies. These three bioactivities have not yet been examined in other members of the Beilschmiedia genus.

## Figures and Tables

**Table 1:** ^1^H (600 MHz) and ^13^C (150 MHz) NMR data of **1** (acetone-*d*_6_) and **2** (CD_3_OD).

position	1		2	
	
	*δ*_C_	*δ*_H_ (*J* in Hz)	*δ*_C_	*δ*_H_ (*J* in Hz)
1	41.6 (CH)	2.36, m	42.0 (CH)	2.41, br dd (12.0, 6.0)
2	31.5 (CH_2_)	1.56, td (12.0, 6.0) 1.65, br dd (12.0, 5.4)	31.0 (CH_2_)	1.57, td (12.0, 6.0) 1.62, br dd (12.0, 5.4)
3	50.5 (CH)	3.05, ddd (13.5, 12.0, 5.4)	54.3 (CH)	2.94, ddd (13.2, 12.0, 5.2)
4	201.6 (C)	-	203.7 (C)	-
5	135.8 (CH)	6.70, d (1.2)	132.3 (CH)	6.54, s
6	151.5 (C)	-	158.5 (C)	-
7	36.2 (CH)	3.51, br s	71.2 (C)	-
8	123.5 (CH)	5.56, ddd (10.2, 3.0, 1.8)	125.9 (CH)	6.01, br d (10.2)
9	131.5 (CH)	5.85, ddd (10.2, 4.2, 3.0)	132.6 (CH)	5.87, dd (10.2, 4.2)
10	35.8 (CH)	2.40, m	35.9 (CH)	2.47, m
11	48.0 (CH)	1.47, m	47.9 (CH)	1.45, m
12	35.2 (CH)	2.87, m	32.9 (CH)	2.97, br dd (8.4, 6.0)
13	44.8 (CH)	2.35, m	51.9 (CH)	2.24, ddd (13.2, 8.4, 1.2)
14	168.3 (C)	-	-	-
1′	38.4 (CH_2_)	1.54, m	38.2 (CH_2_)	1.54, m
2′	28.3 (CH_2_)	1.28, m	28.0 (CH_2_)	1.25, m
3′	31.0 (CH_2_)	1.28, m	30.4 (CH_2_)	1.28, m
4′	33.2 (CH_2_)	1.28, m	33.0 (CH_2_)	1.28, m
5′	23.9 (CH_2_)	1.28, m	23.7 (CH_2_)	1.30, m
6′	15.0 (CH_3_)	0.88, t (7.2)	14.4 (CH_3_)	0.90, t (6.9)

**Table 2:** ^1^H (600 MHz) and ^13^C (150 MHz) NMR data of **3** (CDCl_3_) and **4** (acetone-*d*_6_).

position	3		4	
	
	*δ*_C_	*δ*_H_ (*J* in Hz)	*δ*_C_	*δ*_H_ (*J* in Hz)
1	41.9 (CH)	2.37, m	43.7 (CH)	2.72, br dd (12.0, 5.4)
2	31.8 (CH_2_)	1.28, m 1.99, ddd (14.4, 9.6, 4.8)	41.3 (CH)	2.45, dt (9.0, 5.4)
3	132.3 (CH)	5.50, ddd (15.6, 9.6, 6.0)	41.1 (CH)	1.72, m
4	130.1 (CH)	5.24, ddd (15.6, 9.6, 5.4)	41.2 (CH)	2.25, br t (7.8)
5	31.2 (CH_2_)	2.38, m	41.0 (CH)	2.36, m
6	34.3 (CH_2_)	2.29, ddd (11.4, 5.4, 2.4) 2.22, m	39.9 (CH_2_)	1.63, br d (12.3) 1.87, br dd (12.3, 5.4)
7	173.5 (C)	-	43.4 (CH)	1.89, br dd (9.0, 5.4)
8	-	-	48.2 (CH)	2.91, br dd (8.2, 3.6)
9	70.1 (CH)	4.87, dt (5.6, 2.4)	38.8 (CH)	2.53, m
10	67.2 (CH)	3.96, br d (7.2)	134.2 (CH)	6.19, br t (7.2)
11	40.8 (CH)	2.21, m	131.9 (CH)	6.25, br t (7.2)
12	33.3 (CH)	3.18, br dt (15.6, 7.5)	-	-
13	30.9 (CH)	3.02, m	-	-
14	136.0 (CH)	6.16, dd (10.4, 6.0)	-	-
15	123.9 (CH)	6.25, dd (10.4, 5.6)	-	-
1′	36.3 (CH_2_)	1.47, q (7.5)	43.1 (CH_2_)	2.78, dd (15.6, 7.8) 2.81, br d (7.8)
2′	27.7 (CH_2_)	1.27–1.33, m	136.5 (C)	-
3′	29.5 (CH_2_)	1.27–1.33, m	110.5 (CH)	6.75, d (1.8)
4′	31.9 (CH_2_)	2.24, m	149.2 (C)	-
5′	22.7 (CH_2_)	1.27–1.33, m	147.3 (C)	-
6′	14.1 (CH_3_)	0.90, t (6.9)	109.3 (CH)	6.74, d (7.8)
7′	-	-	123.1 (CH)	6.68, dd (7.8, 1.8)
1″	-	-	155.3 (CH)	6.72, dd (15.6, 8.2)
2″	-	-	121.3 (CH)	5.71, dd (15.6, 0.9)
3″	-	-	168.4 (C)	-
OCH_2_O	-	-	102.3 (CH_2_)	5.94, s

**Table 3:** ^1^H (600 MHz) and ^13^C (150 MHz) NMR data of **6** (CDCl_3_) and **7** (CDCl_3_).

position	6		7	
	
	*δ*_C_	*δ*_H_ (*J* in Hz)	*δ*_C_	*δ*_H_ (*J* in Hz)
1	36.9 (CH_2_)	1.37, br d (12.0) 1.69, dd (12.0, 4.2)	56.9 (CH)	1.65, ddd (10.2, 3.6, 1.8)
2	42.8 (CH)	2.64, d (4.2)	23.2 (CH_2_)	1.33, m 1.66, m
3	147.7 (C)	-	40.8 (CH_2_)	1.56, ddd (15.0, 8.4, 1.8) 1.93, ddd (15.0, 11.1, 2.1)
4	147.5 (CH)	6.60, dd (3.6, 0.6)	75.1 (C)	-
5	71.2 (CH)	4.09, d (3.6)	73.3 (CH)	3.60, br dd (6.0, 2.4)
6	48.7 (C)	-	32.6 (CH_2_)	1.57, m 1.75, m
7	56.9 (C)	-	34.7 (CH_2_)	2.05, m 2.43, ddd (13.2, 9.0, 4.2)
8	36.2 (CH_2_)	1.10, m 1.58, m	151.8 (C)	-
9	38.6 (CH_2_)	1.25, m	42.3 (CH)	2.37, q (10.2)
10	27.1 (CH_2_)	1.32, br dd (6.9, 0.9) 1.54, m	36.0 (CH_2_)	1.58, m 1.74, t (10.2)
11	54.9 (C)	-	34.1 (C)	-
12	26.9 (CH_3_)	1.089, s	21.5 (CH_3_)	1.14, s
13	24.2 (CH_3_)	0.96, s	110.5 (CH_2_)	4.92, d (1.2) 4.94, d (1.2)
14	18.7 (CH_3_)	1.087, s	22.1 (CH_3_)	0.98, s
15	193.3 (CH)	9.54, s	30.1 (CH_3_)	1.00, s
OH	-	-	-	2.07, br s
OH	-	-	-	2.29, br d (3.0)

**Table 4:** Mean E_max_ and IC_50_ of isolates from roots of *B. tsangii* on nitrite production induced by LPS in RAW 264.7 cells.

Compounds	E_max_ (%) a	IC_50_ (μM) b
endiandric acid M (**4**)	97.03 ± 1.30	31.70 ± 0.25
beilschminol B (**5**)	55.62 ± 1.96	95.37 ± 0.52
ursolic acid (**11**)	36.55 ± 4.66	>100
6β-hydroxystigmast-4-en-3-one c	11.69 ± 2.91	>100
*rel*-(7*S*,8*S*,7′*R*,8′*R*)-3,4,5,3′,4′,5′-hexmethoxylignan^c^	52.67 ± 5.05	98.26 ± 0.13
aminoguanidine d (a selective iNOS inhibitor)	80.35 ± 0.26	26.55 ± 0.48
*N*^ω^-nitro-_L_-arginine d (a nonselective iNOS inhibitor)	43.72 ± 0.76	152.46 ± 10.53

a E_max_ indicates mean maximum inhibitory effect, at a concentration of 100 μM, expressed as a percentage inhibition of nitrite production induced by LPS (200 ng/mL) in the presence of vehicle;

b IC_50_ means the concentration producing 50% E_max_. (*n* = 4–6 in each group);

c This compound was reported in Huang *et al.*, 2011 [4];

d positive control.
